# HIV is an independent predictor of aortic stiffness

**DOI:** 10.1186/s12968-014-0057-1

**Published:** 2014-08-16

**Authors:** Oliver J Rider, Mina Asaad, Ntobeko Ntusi, Emma Wainwright, Genevieve Clutton, Gemma Hancock, Rajarshi Banerjee, Alex Pitcher, Katherine Samaras, Kieran Clarke, Stefan Neubauer, Lucy Dorrell, Cameron J Holloway

**Affiliations:** 1 Department of Physiology, Anatomy and Genetics, University of Oxford, Parks Road, Oxford OX1 3PT, UK; 2 Department of Cardiovascular Medicine, University of Oxford Centre for Clinical Magnetic Resonance Research, (OCMR), John Radcliffe Hospital, Oxford OX3 9DU, UK; 3 Oxford NIHR Biomedical Research Centre, University of Oxford, Oxford, UK; 4 St Vincent’s Hospital, Sydney, Australia

**Keywords:** Aorta, Cardiovascular magnetic resonance, HIV

## Abstract

**Background:**

Patients with treated Human Immunodeficiency Virus-1 (HIV) infection are at increased risk of cardiovascular events. Traditionally much of this risk has been attributed to metabolic and anthropometric abnormalities associated with HIV, which are similar to the metabolic syndrome (MS), an established risk factor for cardiovascular mortality. It remains unclear whether treated HIV infection is itself associated with increased risk, via increase vascular stiffness.

**Methods:**

226 subjects (90 with HIV) were divided into 4 groups based on HIV and MS status: 1) HIV-ve/MS-ve, 2) HIV-ve/MS + ve, 3) HIV + ve/MS-ve and 4)HIV + ve/MS + ve. CMR was used to determine aortic pulse wave velocity (PWV) and regional aortic distensibility (AD).

**Results:**

PWV was 11% higher and regional AD up to 14% lower in the HIV + ve/MS-ve group when compared to HIV-ve/MS-ve (p < 0.01 all analyses). PWV and AD in the HIV + ve/MS-ve group was similar to that observed in the HIV-ve/MS + ve group (p > 0.99 all analyses). The HIV + ve/MS + ve group had 32% higher PWV and 30-34% lower AD than the HIV-ve/MS-ve group (all p < 0.001), and 19% higher PWV and up to 31% lower AD than HIV + ve/MS-ve subjects (all p < 0.05). On multivariable regression, age, systolic blood pressure and treated HIV infection were all independent predictors of both PWV and regional AD.

**Conclusion:**

Across multiple measures, treated HIV infection is associated with increased aortic stiffness and is also an independent predictor of both PWV and regional AD. The magnitude of the effect of treated HIV and MS are similar, with additive detrimental effects on central vascular elasticity.

## Background

Effective antiretroviral therapy has markedly improved life expectancy in the HIV-infected population. However, despite immunological modulation, aging with HIV is accompanied by an increased prevalence of metabolic abnormalities, atherosclerotic cardiovascular disease and higher all-cause mortality [1]–[4]. The pathogenesis of this accelerated atherosclerosis in HIV has not been fully elucidated. Even after adjusting for traditional risk factors, which tend to have a higher prevalence in the treated HIV population, elevated rates of coronary disease remain [2]. Recent studies have suggested that viral factors, in addition to traditional vascular risk factors, have independent negative effects, possibly by promoting atherogenic lipid abnormalities [5], monocyte attraction and migration into the vascular intima [6] and a chronic inflammatory state [7],[8], all of which produce an environment for accelerated atherogenesis.

In addition, treated HIV is associated with an increase in the prevalence of the cluster of metabolic risk factors that make up the metabolic syndrome [9]. Metabolic complications appear to result from treatments for HIV and, to some degree, from the effect of viral infection itself [10]–[13]. Whether viral factors amplify the atherogenic effect of traditional metabolic risk factors in HIV-infected individuals remains unclear.

Both aortic pulse wave velocity (PWV) and regional aortic distensibility (AD) are sensitive markers of aortic elastic function. Aortic pulse wave velocity is a clinical measure of arterial stiffness that has not only been shown to be predictive of both cardiovascular events and mortality in multiple population studies, [14],[15] but is also a well-validated surrogate marker of atherosclerosis, corresponding closely to the degree of atherosclerosis as assessed by both computed tomography and post-mortem studies [16]. The aim of this study was to use PWV and AD to investigate 1) if HIV is independently associated with increased arterial stiffness, 2) the magnitude of the detrimental effect of HIV on aortic elastic function when compared to the metabolic syndrome and 3) whether or not the metabolic syndrome and HIV are additive in their detrimental effects.

## Methods

### Study cohort

90 patients with documented HIV infection (13 naive to antiretroviral therapy) and 136 subjects with no history of HIV infection were recruited to this study. Eligibility criteria were age over 18 years, no history or symptoms of cardiovascular disease and no contraindications to cardiovascular magnetic resonance (CMR). All patients with HIV were treated in accordance with national guidelines. Patients with HIV and non-infected subjects were subdivided into those with and without the metabolic syndrome according to the National Cholesterol Education Program-Adult Treatment Panel III (NCEP-ATP III) guidelines [17]. Subjects were diagnosed with the MS when at least 3 of 5 risk determinants increased waist circumference, increased systolic blood pressure (SBP) or diastolic blood pressure (DBP), elevated serum triglycerides, low high-density lipoprotein cholesterol (HDL-C), and impaired fasting glucose (IFG) were present. Subjects were then separated into 4 groups: 1) healthy controls without the MS (HIV-ve/MS-ve), 2) controls with the MS (HIV-ve/MS + ve), 3) patients with HIV without the MS (HIV + ve/MS-ve) and 4) patients with HIV and the MS (HIV + ve/MS + ve). This study was approved by the local ethics committee (ref. 10/H0604/95) and all subjects gave informed written consent prior to participation. Ethics was approved by the Oxford Research Ethics Committee.

#### Anthropometric data

Morning assessments took place after a minimum 10 hour fast. Height and weight were measured using a digital station (Seca, UK) and used to calculate body mass index (BMI, mass (kg)/height (m)^2^). Waist circumferences were measured in the standing position at the level of the umbilicus with the average of three measurements taken. Data on duration of HIV infection, nadir CD4 count and Highly Active Antiretroviral Therapy (HAART) were obtained from the treating physicians with the consent of the patients.

A fasting blood sample was obtained for measurement of plasma glucose, free fatty acids, hs-CRP, cholesterol and hs-CRP and were analysed by a commercial hospital laboratory. Insulin was measured using ELISA (Mercodia AB, Uppsala, Sweden). To calculate the homeostasis model assessment for insulin resistance (HOMA-IR) the following formula was used: fasting insulin (pmol/l) × fasting glucose (mM/l)/135 and used as a measure of insulin resistance [18].

### CMR protocol for aortic distensibility

All vascular imaging was performed at 1.5-Tesla (Avanto, Siemens Medical Solutions, Erlangen, Germany). Based on sagittal-oblique (candy cane) pilot images aligned with the aortic arch, thoracic aortic cine images were acquired in transverse planes at 3 levels as previously described: [19] the crossing of the pulmonary arch through 1) the ascending thoracic aorta (Ao), 2) proximal descending thoracic aorta (PDA) and 3) 12 cm below the PDA level piloted perpendicular to the orientation of the aorta (distal descending aorta, DDA). A brachial artery blood pressure was recorded during image acquisition to provide pulse pressure [20].

#### CMR protocol for aortic pulse wave velocity

**Figure 1:**
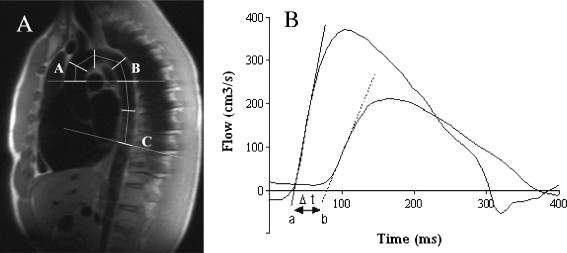
**Calculating pulse wave velocity.A**. Distance Δx was taken as the total distance between A (ascending aorta) and C (abdominal aorta). This was calculated using the sum of the distances between the centre points of lines drawn at 45°, 90° and 135° to the scan level. **B**. Aortic flow/time curves used to calculate the arrival times of aortic pulse waveform. Δt represents the time (m/s) between the intercepts (b-a) of the tangents to the curve at the half maximal point of flow in the ascending aorta (a) and the abdominal aorta (b).

To measure aortic PWV, images were acquired using a free-breathing, retrospectively ECG-gated, spoiled gradient echo sequence. Velocity-encoding gradient for phase contrast was applied to measure through-plane flow in the ascending aorta at the same three levels used for aortic distensibility. Oblique sagittal images were used to calculate the distance between the two imaging levels as previously described (Figure 1) [21].

#### CMR protocols for visceral fat mass

To measure visceral fat mass, a single breath-hold, single-slice, water-suppressed T1-weighted turbo spin echo sequence centred on L5 was acquired as previously described [22].

#### Image analysis

Image analysis was performed using Siemens analytical software (Argus ©, Siemens Medical Solutions, Erlangen, Germany). To analyse aortic PWV, flow images were manually contoured [23] and aortic PWV was determined as Δx/Δt (m/s), where Δx is the aortic distance between two imaging levels and Δt is time delay between the arrival of the foot of the pulse wave between these imaging levels (Figure 1) [24]. The arrival of the foot of the pulse wave was taken as the intersection of the tangent (determined by a least squares method of the four flow points around the half maximal point of flow) of the upslope of the flow curve and the x (time) axis as previously described [19],[24]. To assess visceral fat mass, transverse slices were manually countered as previously described [25].

#### Statistical analysis

All statistical analyses were performed using SPSS statistical software (version 19.0; SPSS Inc., Chicago, Ill, USA). Normality of distribution was assessed using the Kolmogorov-Smirnov test. Normally distributed data are presented as mean ± standard deviation, non-normally distributed data as median (interquartile range). Comparisons between normally distributed data in the 4 groups identified above were performed with one-way analysis of variance with post-hoc Bonferroni correction. Bivariate correlations for all subjects were calculated using Pearson correlation coefficients. Significance was assumed at a probability value of p < 0.05. Variables with p < 0.05 and the strongest relationship with aortic pulse wave velocity and AD were then included as independent variables in forced entry stepwise multiple regression analysis with aortic PWV and AD as the dependent variables.

## Results

### Baseline characteristics of the study populations

Anthropometric data for the study group when divided into the four groups is shown in Table 1. The immunological, virological and HAART status of patients with HIV are presented in Table 2.

**Table 1:** Anthropometric characteristics of the study population

	**HIV-ve/MS-ve (n = 92)**	**HIV-ve/MS + ve (n = 44)**	**HIV + ve/MS-ve (n = 73)**	**HIV + ve/MS + ve (n = 17)**	**P value from analysis of variance**
**Age (years)**	45 ± 10	49 ± 11	44 ± 10	48 ± 8	0.12
**BMI (kg/m**^**2**^**)**	25 ± 4	33 ± 7*	25 ± 4^Φ^	30 ± 6*^ψ^	<0.001
**Visceral Fat (cm**^**2**^**)**	71 ± 39	160 ± 71*	86 ± 49^Φ^	120 ± 66*	<0.001
	0.9 ± 0.5	1.7 ± 0.8*	1.3 ± 1.0	2.4 ± 1.7*^ψ^	<0.001
**Cholesterol (mmol/L)**	4.8 ± 0.9	5.1 ± 1.2	4.5 ± 1.4	4.6 ± 1.4	0.110
**HDL-C (mmol/L)**	1.6 ± 0.4	1.1 ± 0.3*	1.2 ± 0.4*	1.0 ± 0.4*	<0.001
**Glucose (mmol/L)**	4.8 ± 0.5	5.4 ± 0.8*	5.0 ± 0.8	6.0 ± 1.3*^Φψ^	<0.001
**HOMA-IR**	1.1 ± 1.0	3.5 ± 2.7*	1.4 ± 1.3^Φ^	4.6 ± 6.1*^ψ^	<0.001
**hs-CRP (mg/L)**	0.7 ± 1.6	3.1 ± 4.0*	2.2 ± 2.7*	4.6 ± 4.5*^ψ^	<0.001
**SBP (mmHg)**	117 ± 12	130 ± 14*	118 ± 16^Φ^	138 ± 16*^ψ^	<0.001
**DBP (mmHg)**	73 ± 9	80 ± 8*	75 ± 9^Φ^	85 ± 8*^ψ^	<0.001
**Waist circumference (cm)**	88 ± 14	110 ± 17*	89 ± 11^Φ^	106 ± 16*^ψ^	<0.001
**Smoking (%)**	32	32	37	41	0.799
**PWV (m/s)**	5.6 ± 1.0	6.7 ± 1.7*	6.2 ± 1.9*	7.4 ± 2.4*^ψ^	<0.001

*p < 0.05 versus HIV-ve/MS-ve.

^Φ^p < 0.05 versus HIV-ve/MS + ve.

^ψ^p < 0.05 versus HIV + ve/MS-ve.

**Table 2:** HIV specific data for the HIV + ve/MS-ve and HIV + ve/MS + ve groups, data presented as median (Interquartile range)

	**HIV + ve/MS-ve**		**HIV + ve/MS + ve**	
	**On HAART (n = 61)**	**No HAAR (n = 12)**	**On HAART (n = 16)**	**No HAART (n = 1)**
**Duration of HIV infection (years)**	6 (7)	2 (4)	8 (9)	6
**Time on HAART (years)**	3 (5)	-	6 (7)	-
**Nadir CD4 cell count (10**^**6**^**/L)**	190 (170)	560 (425)	170 (190)	300
**Current CD4 cell count (10**^**6**^**/L)**	515 (218)	640 (400)	550 (255)	300
**HIV viral load (copies/mL)**	10 (10)	896 (17,613)	10 (10)	55,095
**Protease inhibitors (%)**	21	-	41	-
**NRTI (%)**	81	-	94	-
**NNRTI (%)**	61	-	53	-

### The effect of HIV on aortic elastic function

**Figure 2:**
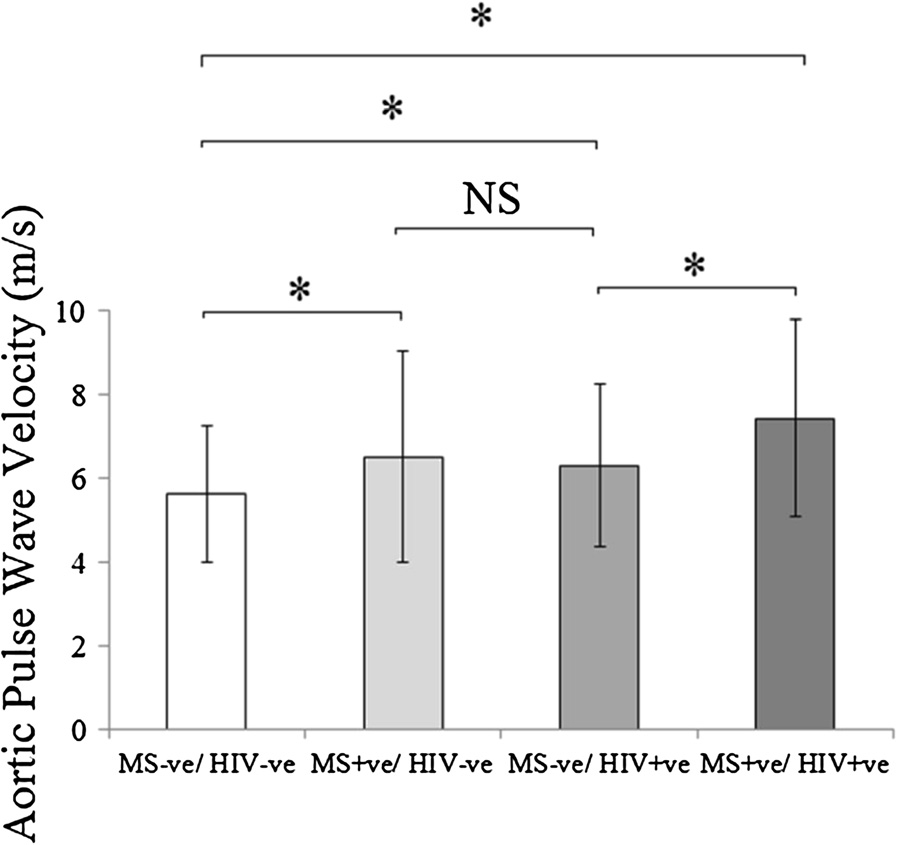
The effect of HIV and the metabolic syndrome on aortic pulse wave velocity.

**Figure 3:**
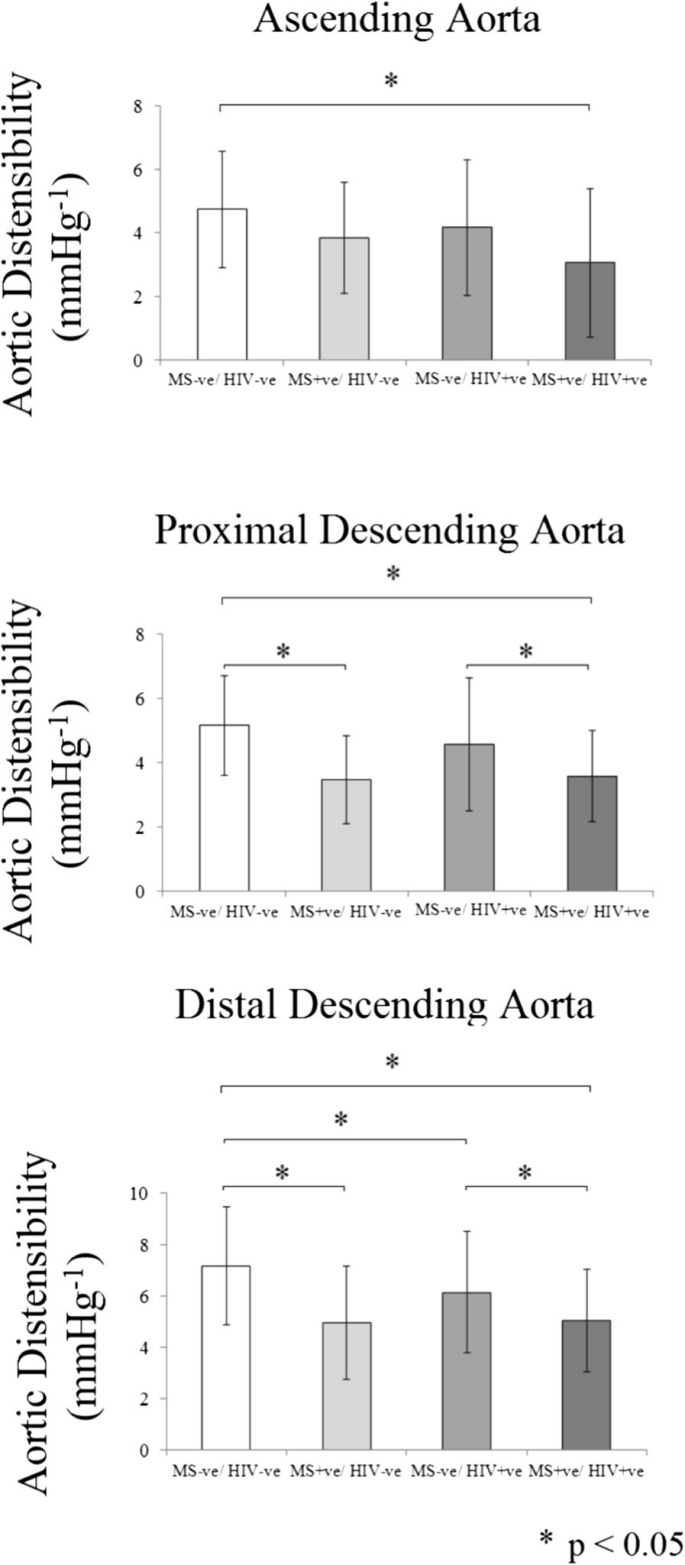
**The effect of HIV and the metabolic syndrome on aortic distensibility.** For Clarity Only Differences in Distensibility Reaching a Statistical Significance of p < 0.05 are Highlighted.

To isolate the effect of HIV alone on aortic PWV and AD, patients with HIV but without the metabolic syndrome (HIV + ve/MS-ve, n = 73) were compared to non-infected subjects without the metabolic syndrome (HIV-ve/MS-ve, n = 92). The two groups were well matched for age, blood pressure, BMI, visceral fat, waist circumference, triglycerides, cholesterol, glucose, HOMA-IR and smoking status (Table 1). In agreement with the published literature, HIV infection was associated with reduced HDL cholesterol, a higher prevalence of smoking and an elevated high sensitivity C- reactive protein level (hs-CRP, Table 1). PWV was 11% higher in the HIV + ve/MS-ve group when compared to the HIV-ve/MS-ve group (6.2 ± 1.9 vs 5.6 ± 1.9 m/s, p = 0.008, Figure 2) and aortic distensibility reduced (PDA by 12%, AA by 14%, both p < 0.01, Figure 3). Put together this suggests that HIV alone causes increased aortic stiffness. Of note, there was no difference between regional AD and PWV when comparing HAART naïve (n = 13) to HAART treated (n = 77) subjects (p >0.2 for all analyses).

### Comparing the effects of HIV and the metabolic syndrome on aortic elastic function

In order to compare the effect of HIV and the Metabolic syndrome on PWV and AD, subjects with the metabolic syndrome but without a history of HIV infection (HIV-ve/MS + ve, n = 44) were compared to patients with HIV infection without the metabolic syndrome (HIV + ve/MS-ve, n = 73). As expected, the group with metabolic syndrome had higher blood pressures, BMI, visceral fat and waist circumference when compared to the group without metabolic syndrome (Table 1). Interestingly, despite this, both the HIV-ve/MS + ve and the HIV + ve/MS-ve groups had similar PWV (6.7 ± 1.9 vs 6.2 ± 1.9 m/s p = 0.94), which were 16-20% higher than the HIV-ve/MS-ve group (p < 0.05 for both analyses). A similar pattern was seen comparing distensibility in the HIV-ve/MS + ve and the HIV + ve/MS-ve with aortic distensibility measures at all three levels of the aorta being similar across the two groups (Figure 3). This suggests HIV and the metabolic syndrome have a similar magnitude of effect on aortic elastic function.

### Investigating an additive effect of treated HIV and the metabolic syndrome

To investigate whether HIV and the metabolic syndrome are additive in their negative effects on PWV, patients with HIV infection without the metabolic syndrome (HIV + ve/MS-ve, n = 73) were compared to subjects with both HIV infection and the metabolic syndrome (HIV + ve/MS + ve, n = 17). As would be expected the HIV + ve/MS + ve group had increased BMI, SBP, DBP, waist circumference, HOMA-IR, glucose and hs-CRP when compared to the HIV+/MS-ve group (Table 1). Again, as expected, both the HIV + ve/MS-ve and the HIV + ve/MS + ve groups had higher PWV than the HIV-ve/MS-ve group (PWV by 15% and 38% respectively p < 0.05 for both analyses) In agreement with this, both the HIV + ve/MS-ve and the HIV + ve/MS + ve groups had lower aortic distensibility than the HIV-ve/MS-ve group (Ao by 16% and 44%, PDA by 20% and 45%, DDA by 18% and 38%, respectively all p < 0.05). Importantly, PWV in the HIV + ve/MS + ve group was 19% higher and aortic distensibility 31%, lower (= 0.011, Figure 3) than that recorded in the HIV + ve/MS-ve group (7.4 ± 2.4 vs 6.2 ± 1.9 m/s, p < 0.05). Put together, this suggests an additive detrimental effect of HIV and the metabolic syndrome on aortic elastic function.

### Determinants of aortic PWV in study population

In order to investigate the associations between PWV and other study parameters of the metabolic syndrome and HIV infection, all 226 subjects were included in this analysis. Associations between anthropometric, metabolic and HIV-related characteristics and aortic PWV are presented in Table 3. In agreement with the published literature, aortic PWV was positively correlated with age, BMI, systolic blood pressure, diastolic blood pressure, waist circumference, visceral fat mass, fasting serum glucose and hs-CRP levels. Interestingly, HIV infection per se, current CD4 count and smoking status were also positively correlated with PWV. Importantly, there was no association between duration of HAART, antiretroviral class, viral load or nadir CD4 count and aortic PWV. To investigate if there were any independent predictors of pulse wave velocity, multivariable regression was used as described above. The strongest stepwise multivariable model in this study consisted of aortic pulse wave velocity as the dependent variable and age, SBP, waist circumference, fasting serum glucose, hs-CRP and HIV infection as independent variables. This showed that age (β = 0.065, p < 0.001), SBP (β = 0.017, p = 0.043) and HIV infection (β = 0.53, p = 0.037) were all independent predictors of aortic PWV in this study group (overall R^2^ of the model = 0.33, p < 0.001). This suggests that along with age and blood pressure, HIV infection is independently associated with greater aortic stiffness.

**Table 3:** Associations between PWV and study characteristics of HIV and the metabolic syndrome

			**Aortic distensibility (mmHg**^**-1**^**)**					
	**Pulse wave velocity**		**Ascending**		**Proximal descending**		**Abdominal**	
	**r**	**p**	**r**	**p**	**r**	**p**	**r**	**p**
**Age (years)**	0.52	<0.001	−0.41	<0.001	−0.37	<0.001	−0.29	<0.001
**BMI (kg/m**^**2**^**)**	0.20	0.043	−0.15	0.11	−0.23	<0.001	−0.22	<0.001
**Visceral Fat (cm**^**2**^**)**	0.24	0.008	−0.09	0.28	−0.25	<0.001	−0.22	0.01
**Glucose (mmol/l)**	0.15	0.049	−0.13	0.12	−0.24	<0.001	−0.17	0.03
**Triglycerides (mmol/L)**	0.13	0.09	−0.07	0.37	−0.20	0.01	−0.24	<0.001
**HDL-C (mmol/L)**	−0.14	0.07	0.01	0.99	0.08	0.34	0.09	0.26
**hsCRP (mg/L)**	0.33	<0.001	−0.14	0.01	−0.30	<0.001	−0.26	<0.001
**SBP (mmHg)**	−0.27	<0.001	−0.29	<0.001	−0.32	<0.001	−0.36	<0.001
**DBP (mmHg)**	0.20	<0.001	−0.17	<0.02	−0.21	<0.001	−0.23	<0.001
**Waist circumference (cm)**	0.19	0.02	−0.15	0.08	−0.21	<0.001	−0.31	<0.001
**Cholesterol (mmol/L)**	0.13	0.87	−0.12	0.14	−0.16	0.04	−0.11	0.18
**HOMA-IR**	0.09	0.39	−0.08	0.35	−0.15	0.06	−0.15	0.05
**Past or present smoking**	0.28	<0.001	−0.17	0.03	−0.18	0.03	−0.10	0.20
**Viral load (copies/mL)**	0.06	0.56	−0.12	0.30	−0.12	0.29	−0.04	0.69
**Nadir CD4 count (10**^**6**^**/L)**	−0.13	0.30	−0.06	0.62	0.09	0.42	0.16	0.17
**Current CD4 count (10**^**6**^**/L)**	0.26	0.014	−0.14	0.23	−0.02	0.83	−0.05	0.67
**PIs**	0.06	0.51	0.11	0.23	0.03	0.75	−0.11	0.24
**NRTIs**	0.11	0.22	−0.10	0.27	−0.17	0.08	−0.22	0.02
**NNRTIs**	0.05	0.58	−0.18	0.06	−0.18	0.05	−0.12	0.18
**Duration of HAART (years)**	0.20	0.09	−0.12	0.36	−0.07	0.57	−0.10	0.42
**HIV infection**	0.23	0.001	−0.21	0.01	−0.25	<0.001	−0.21	0.01

### Determinants of regional aortic distensibility

In order to investigate the associations between regional AD and other study parameters of the metabolic syndrome and HIV infection, all 203 subjects were included in this analysis. Associations between anthropometric, metabolic and HIV-related characteristics and aortic AD are presented in Table 3. In agreement with the published literature, aortic distensibility was negatively correlated with age, systolic blood pressure, diastolic blood pressure, visceral fat mass, fasting serum glucose and hs-CRP levels at all levels measured. Importantly, HIV infection was also associated with reduced distensibility at all three levels measured. In addition, as expected, BMI, waist circumference and triglyceride levels were negatively correlated with proximal descending and negatively with distal descending thoracic aortic distensibility (Table 3). In a similar pattern to that seen with PWV, there was no association between duration of HAART, antiretroviral class, viral load or nadir CD4 count and aortic AD. To investigate if there were any independent predictors of pulse wave velocity, multivariable regression was used as described above. The strongest stepwise multivariable models in this study consisted of aortic distensibility (Ao, PDA, DDA) as the dependent variables and age, BMI, SBP, fasting serum glucose, presence of the metabolic syndrome, smoking status and HIV infection as independent variables. This showed that age (Ao β = −0.108, PDA β = −0.05, DDA β = −0.06, all p < 0.001), SBP (Ao β = −0.09, PDA β = −0.03, DDA β = −0.04, all p < 0.01) and HIV infection (Ao β = −0.41, PDA β = −0.08, DDA β = −1.2, all p <0.02) were all independent predictors of aortic AD at all levels measured. In addition, again as expected BMI was an independent predictor of DDA (β = − 0.12, p = 0.001). Overall R^2^ of the models; Ao = 0.48, PDA 0.43, DDA 0.39, all p < 0.001. This suggests that along with age, blood pressure and BMI, HIV infection is independently associated with increased aortic stiffness.

## Discussion

This study has shown that, after matching for potential confounders, HIV infection is independently associated with increased aortic PWV and decreased aortic distensibility, both sensitive markers of reduced aortic elastic function. In addition HIV infection is an independent predictor of both increased pulse wave velocity and decreased aortic distensibility, clinical measures of aortic stiffness linked to increased cardiovascular mortality. The size of this detrimental effect is similar to that seen with the metabolic syndrome, a powerful cardiovascular risk factor. We have shown that HIV and the metabolic syndrome are additive in their negative effects on PWV and aortic distensibility, suggesting that both are risk factors that act in different ways to impair vascular elasticity. The mechanism of vascular alterations in patients with HIV may be secondary to direct effects of the HIV virus on vascular function, including direct alteration in endothelial function, inflammation, and modification of aortic wall vascular smooth muscle cell behaviour and extra-cellular matrix composition.

### Chronic inflammation

There is significant evidence that HIV infection is associated with chronic systemic and vascular inflammation even in patients on stable HAART with preserved CD4 counts and HIV RNA levels < 50 copies/ml, [8],[26] and these patients also have a greater burden of subclinical atherosclerosis [7]. Elevations in inflammatory markers, such as CRP, are associated with both increased arterial stiffness [27] and a higher risk of mortality [28]. In support of a chronic inflammatory HIV-related vasculopathy explaining our findings, a recent 18 F-FDG-PET study demonstrated signs of increased arterial inflammation in treated subjects with HIV [8]. Moreover, hsCRP was not only elevated in patients with HIV in this study but was also positively correlated to aortic PWV and negatively correlated to AD at all three levels, and furthermore was strongly correlated with visceral fat, which is thought to play a central role in this inflammatory process, with macrophage release of IL-6 and TNF-α [29] both which are associated with increased PWV. This may suggest a role for elevated visceral fat in vascular elastic dysfunction in treated HIV [30].

This study has demonstrated impaired vascular function in HIV subjects, independent of traditional risk factors, and, together with other studies of contemporary cohorts of patients with treated HIV, suggest that chronic inflammation in HIV infection may partially underlie the reduced vascular function and premature atherosclerosis. Prospective studies to reduce chronic inflammation beyond HAART are required to investigate whether or not this improves vascular inflammation and function and ultimately reduces atherosclerotic risk in patients with HIV.

### Endothelial dysfunction in HIV

Endothelial dysfunction is a common feature of atherosclerosis [31]. There is a growing body of evidence suggesting HIV itself may have deleterious effects on endothelial function. Patients with HIV (but without the metabolic syndrome) have been shown to have reduced vascular reactivity in a pattern similar to that seen in type 2 diabetes mellitus [32]. The direct effect of HIV on endothelial biology has been extensively investigated. As a result of increased secretion of monocyte chemo-attractant protein 1 [33] and increased expression of the adhesion molecules, vascular cell adhesion molecule-1 (VCAM-1) intercellular adhesion molecule 1 and E-selectin [6], vascular endothelium from patients with HIV have increased monocyte adherence and monocyte migration into the vascular intima during atherosclerotic plaque development [34]. In addition, several HIV proteins, notably Tat and gp120 are themselves associated with endothelial cell activation and increased expression cell adhesion molecules [35]. Put together with the evidence that HIV infection affects lipid processing [5] and is associated with lower HDL cholesterol, lower apolipoprotein B levels (in advanced disease) [36] and smaller LDL cholesterol particles, this environment provides an atherogenic milieu [37]. Although studies have now shown that HAART may to some extent reduce endothelial activation, it does not appear to completely ameliorate the effects of HIV infection, suggesting that even during complete virological suppression, endothelial dysfunction persists and continues to promote atherosclerosis (and excess cardiovascular risk) in HIV [38],[39].

### The additive effect of treated HIV infection and the metabolic syndrome on arterial stiffness

The detrimental effect of the metabolic syndrome on aortic stiffness is well established [40]–[42]. This study has not only shown that treated HIV infection has a similar sized effect on aortic stiffness to the metabolic syndrome, but has also shown that the combination of HIV and the metabolic syndrome are additive in their negative effects. This suggests that patients with both are likely to be at even higher risk of cardiovascular events. As hs-CRP was significantly higher in HIV subjects with the MS compared to HIV subjects without the MS, and CRP has been shown to be a marker of excess risk, [41] inflammation is a potential unifying mechanism that may underlie the pathogenesis of vascular disease in both groups.

### Study limitations

Due to the observational nature of this study, it is not possible to confirm causality or mechanisms which might underlie the increased aortic PWV and decreased aortic distensibility in patients with HIV. The study was not powered to detect differences in HAART-naïve and treated subjects. Nor was it possible to determine the effects of individual anti-retroviral medications on vascular function. Given the beneficial effect of antiretroviral treatments on the vasculature and improved markers of vascular function that are observed after commencement of antiretroviral therapy [38],[43], antiretroviral agents are an unlikely cause of the vascular dysfunction. Nevertheless, longitudinal studies are needed to investigate this further. However, by including both HAART naïve and HAART treated patients this study provides a representative dataset of HIV infected subjects in the UK.

Distensibility was calculated using pulse pressure measurements taken from brachial blood pressure rather than central blood pressure recordings. Although we accept central pressure recording may have changed the absolute aortic distensibility measures, given the magnitude of the changes the DDA segment (i.e. up to 45% change in distensibility), the pattern of change, i.e. the main finding of this paper would not be expected to be affected.

## Conclusion

Given the increase in cardiovascular events and cardiovascular related mortality in HIV-infected patients treated with effective antiretroviral therapy, identifying those at higher cardiovascular risk is of great clinical importance. We have shown that patients with treated HIV have reduced vascular function, which is independent of the metabolic syndrome and other traditional cardiovascular risk factors, suggesting a virus-related aetiology, such as chronic inflammation. Importantly we have shown that the magnitude of the effect of treated HIV infection is the same as that of the metabolic syndrome, a well-established and powerful predictor of cardiovascular mortality. Given the additive effect of HIV and the metabolic syndrome on reducing vascular function, aggressive management of both HIV and the metabolic syndrome are likely needed to reduce the high incidence of vascular disease in this population.

## Competing interests

The authors declare that they have no competing interests.

## Authors’ contributions

OR, SN, CH, KC, LD made substantial contributions to conception and design of the study. CJH, NN, RB, AP, OR acquired the CMR data. EW, GH, LD acquired the HIV data. OR, MA analysed and interpreted the data. OR, MA drafted the manuscript. LD, GC, KS, OR, CH revised the manuscript critically for important intellectual content. All authors read and approved the final manuscript.

## References

[B1] Sterne JA Hernan MA Ledergerber B Tilling K Weber R Sendi P Rickenbach M Robins JM Egger M Long-term effectiveness of potent antiretroviral therapy in preventing AIDS and death: a prospective cohort study Lancet 2005 366 378 384 10.1016/S0140-6736(05)67022-5 16054937

[B2] Triant VA Lee H Hadigan C Grinspoon SK Increased acute myocardial infarction rates and cardiovascular risk factors among patients with human immunodeficiency virus disease J Clin Endocrinol Metab 2007 92 2506 2512 10.1210/jc.2006-2190 17456578 PMC2763385

[B3] Grunfeld C Delaney JAC Wanke C Currier JS Scherzer R Biggs ML Tien PC Shlipak MG Sidney S Polak JF O'Leary D Bacchetti P Kronmal RA Cha SFRM Preclinical atherosclerosis due to HIV infection: carotid intima-medial thickness measurements from the FRAM study AIDS 2009 23 1841 1849 10.1097/QAD.0b013e32832d3b85 19455012 PMC3156613

[B4] Tseng ZH Secemsky EA Dowdy D Vittinghoff E Moyers B Wong JK Havlir DV Hsue PY Sudden cardiac death in patients with human immunodeficiency virus infection J Am Coll Cardiol 2012 59 1891 1896 10.1016/j.jacc.2012.02.024 22595409 PMC3356565

[B5] Riddler SA Smit E Cole SR Li R Chmiel JS Dobs A Palella F Visscher B Evans R Kingsley LA Impact of HIV infection and HAART on serum lipids in men JAMA 2003 289 2978 2982 10.1001/jama.289.22.2978 12799406

[B6] Fisher SD Miller TL Lipshultz SE Impact of HIV and highly active antiretroviral therapy on leukocyte adhesion molecules, arterial inflammation, dyslipidemia, and atherosclerosis Atherosclerosis 2006 185 1 11 10.1016/j.atherosclerosis.2005.09.025 16297390

[B7] Hsue PY Hunt PW Schnell A Kalapus SC Hoh R Ganz P Martin JN Deeks SG Role of viral replication, antiretroviral therapy, and immunodeficiency in HIV-associated atherosclerosis AIDS 2009 23 1059 1067 10.1097/QAD.0b013e32832b514b 19390417 PMC2691772

[B8] Subramanian S Tawakol A Burdo TH Abbara S Wei J Vijayakumar J Corsini E Abdelbaky A Zanni MV Hoffmann U Williams KC Lo J Grinspoon SK Arterial inflammation in patients with HIV JAMA 2012 308 379 386 10.1001/jama.2012.6698 22820791 PMC3724172

[B9] Samaras K Prevalence and pathogenesis of diabetes mellitus in HIV-1 infection treated with combined antiretroviral therapy J Acquir Immune Defic Syndr 2009 50 499 505 10.1097/QAI.0b013e31819c291b 19223782

[B10] Badiou S De Boever CM Dupuy AM Baillat V Cristol JP Reynes J Small dense LDL and atherogenic lipid profile in HIV-positive adults: influence of lopinavir/ritonavir-containing regimen AIDS 2003 17 772 774 10.1097/00002030-200303280-00023 12646808

[B11] Sabin CA Worm SW Weber R Reiss P El-Sadr W Dabis F De Wit S Law M D’Arminio Monforte A Friis-Moller N Kirk O Pradier C Weller I Phillips AN Lundgren JD Use of nucleoside reverse transcriptase inhibitors and risk of myocardial infarction in HIV-infected patients enrolled in the D:A:D study: a multi-cohort collaboration Lancet 2008 371 1417 1426 10.1016/S0140-6736(08)60423-7 18387667 PMC2688660

[B12] Friis-Moller N Sabin CA Weber R d’Arminio Monforte A El-Sadr WM Reiss P Thiebaut R Morfeldt L De Wit S Pradier C Calvo G Law MG Kirk O Phillips AN Lundgren JD Combination antiretroviral therapy and the risk of myocardial infarction N Engl J Med 2003 349 1993 2003 10.1056/NEJMoa030218 14627784

[B13] Klein D Hurley LB Quesenberry CP Jr Sidney S Do protease inhibitors increase the risk for coronary heart disease in patients with HIV-1 infection? J Acquir Immune Defic Syndr 2002 30 471 477 10.1097/00126334-200208150-00002 12154337

[B14] Sutton-Tyrrell K Newman A Simonsick EM Havlik R Pahor M Lakatta E Spurgeon H Vaitkevicius P Aortic stiffness is associated with visceral adiposity in older adults enrolled in the study of health, aging, and body composition Hypertension 2001 38 429 433 10.1161/01.HYP.38.3.429 11566917

[B15] Mattace-Raso FU van der Cammen TJ Hofman A van Popele NM Bos ML Schalekamp MA Asmar R Reneman RS Hoeks AP Breteler MM Witteman JC Arterial stiffness and risk of coronary heart disease and stroke: the Rotterdam Study Circulation 2006 113 657 663 10.1161/CIRCULATIONAHA.105.555235 16461838

[B16] Taniwaki H Kawagishi T Emoto M Shoji T Kanda H Maekawa K Nishizawa Y Morii H Correlation between the intima-media thickness of the carotid artery and aortic pulse-wave velocity in patients with type 2 diabetes. Vessel wall properties in type 2 diabetes Diabetes Care 1999 22 1851 1857 10.2337/diacare.22.11.1851 10546019

[B17] Expert Panel on Detection, Evaluation, and Treatment of High Blood Cholesterol in Adults (Adult Treatment Panel III) final report Circulation 2002 106 3143 3421 12485966

[B18] Matthews DR Hosker JP Rudenski AS Naylor BA Treacher DF Turner RC Homeostasis model assessment: insulin resistance and beta-cell function from fasting plasma glucose and insulin concentrations in man Diabetologia 1985 28 412 419 10.1007/BF00280883 3899825

[B19] Wiesmann F Petersen SE Leeson PM Francis JM Robson MD Wang Q Choudhury R Channon KM Neubauer S Global impairment of brachial, carotid, and aortic vascular function in young smokers: direct quantification by high-resolution magnetic resonance imaging J Am Coll Cardiol 2004 44 2056 2064 10.1016/j.jacc.2004.08.033 15542292

[B20] Rider OJ Holloway CJ Emmanuel Y Bloch E Clarke K Neubauer S Increasing plasma free fatty acids in healthy subjects induces aortic distensibility changes seen in obesity Circ Cardiovasc Imaging 2012 5 367 375 10.1161/CIRCIMAGING.111.971804 22492484

[B21] Lewandowski AJ Lazdam M Davis E Kylintireas I Diesch J Francis J Neubauer S Singhal A Lucas A Kelly B Leeson P Short-term exposure to exogenous lipids in premature infants and long-term changes in aortic and cardiac function Arterioscler Thromb Vasc Biol 2011 31 2125 2135 10.1161/ATVBAHA.111.227298 21817105

[B22] Rider OJ Francis JM Ali MK Petersen SE Robinson M Robson MD Byrne JP Clarke K Neubauer S Beneficial cardiovascular effects of bariatric surgical and dietary weight loss in obesity J Am Coll Cardiol 2009 54 718 726 10.1016/j.jacc.2009.02.086 19679250

[B23] Leeson CP Robinson M Francis JM Robson MD Channon KM Neubauer S Wiesmann F Cardiovascular magnetic resonance imaging for non-invasive assessment of vascular function: validation against ultrasound J Cardiovasc Magn Reson 2006 8 381 387 10.1080/10976640500526993 16669182

[B24] Rider OJ Tayal U Francis JM Ali MK Robinson MR Byrne JP Clarke K Neubauer S The effect of obesity and weight loss on aortic pulse wave velocity as assessed by magnetic resonance imaging Obesity 2010 18 2311 2316 10.1038/oby.2010.64 20360756

[B25] Rider OJ, Francis JM, Ali MK, Byrne J, Clarke K, Neubauer S, Petersen SE. **Determinants of left ventricular mass in obesity; a cardiovascular magnetic resonance study.** *J Cardiovasc Magn Reson.* 2009; **11:**9. 10.1186/1532-429X-11-9 PMC2680851 19393079

[B26] Kuller LH, Tracy R, Belloso W, De Wit S, Drummond F, Lane HC, Ledergerber B, Lundgren J, Neuhaus J, Nixon D, Paton NI, Neaton JD, Group ISS. **Inflammatory and coagulation biomarkers and mortality in patients with HIV infection.** *PLoS Med.* 2008; **5:**e203. 10.1371/journal.pmed.0050203 PMC2570418 18942885

[B27] Yasmin MECM Wallace S Mackenzie IS Cockcroft JR Wilkinson IB C-reactive protein is associated with arterial stiffness in apparently healthy individuals Arterioscler Thromb Vasc Biol 2004 24 969 974 10.1161/01.ATV.zhq0504.0173 15001456

[B28] Tuomisto K Jousilahti P Sundvall J Pajunen P Salomaa V C-reactive protein, interleukin-6 and tumor necrosis factor alpha as predictors of incident coronary and cardiovascular events and total mortality. A population-based, prospective study Thromb Haemost 2006 95 511 518 16525580 10.1160/TH05-08-0571

[B29] Van Gaal LF Mertens IL De Block CE Mechanisms linking obesity with cardiovascular disease Nature 2006 444 875 880 10.1038/nature05487 17167476

[B30] Pauletto P Rattazzi M Inflammation and hypertension: the search for a link Nephrol Dial Transplant 2006 21 850 853 10.1093/ndt/gfl019 16464884

[B31] Shimokawa H Primary endothelial dysfunction: atherosclerosis J Mol Cell Cardiol 1999 31 23 37 10.1006/jmcc.1998.0841 10072713

[B32] van Wijk JP de Koning EJ Cabezas MC Joven J op’t Roodt J Rabelink TJ Hoepelman AM Functional and structural markers of atherosclerosis in human immunodeficiency virus-infected patients J Am Coll Cardiol 2006 47 1117 1123 10.1016/j.jacc.2005.09.073 16545639

[B33] Park IW Wang JF Groopman JE HIV-1 Tat promotes monocyte chemoattractant protein-1 secretion followed by transmigration of monocytes Blood 2001 97 352 358 10.1182/blood.V97.2.352 11154208

[B34] Zietz C Hotz B Sturzl M Rauch E Penning R Lohrs U Aortic endothelium in HIV-1 infection: chronic injury, activation, and increased leukocyte adherence Am J Pathol 1996 149 1887 1898 8952525 PMC1865334

[B35] Mu H Chai H Lin PH Yao Q Chen C Current update on HIV-associated vascular disease and endothelial dysfunction World J Surg 2007 31 632 643 10.1007/s00268-006-0730-0 17372667

[B36] Shahmanesh M Das S Stolinski M Shojaee-Moradie F Jackson NC Jefferson W Cramb R Nightingale P Umpleby AM Antiretroviral treatment reduces very-low-density lipoprotein and intermediate-density lipoprotein apolipoprotein B fractional catabolic rate in human immunodeficiency virus-infected patients with mild dyslipidemia J Clin Endocrinol Metab 2005 90 755 760 10.1210/jc.2004-1273 15522931

[B37] Mujawar Z, Rose H, Morrow MP, Pushkarsky T, Dubrovsky L, Mukhamedova N, Fu Y, Dart A, Orenstein JM, Bobryshev YV, Bukrinsky M, Sviridov D. **Human immunodeficiency virus impairs reverse cholesterol transport from macrophages.** *PLoS Biol.* 2006; **4:**e365. 10.1371/journal.pbio.0040365 PMC1629034 17076584

[B38] van Vonderen MG Hassink EA van Agtmael MA Stehouwer CD Danner SA Reiss P Smulders Y Increase in carotid artery intima-media thickness and arterial stiffness but improvement in several markers of endothelial function after initiation of antiretroviral therapy J Infect Dis 2009 199 1186 1194 10.1086/597475 19275490

[B39] Wolf K Tsakiris DA Weber R Erb P Battegay M Swiss HIVCS Antiretroviral therapy reduces markers of endothelial and coagulation activation in patients infected with human immunodeficiency virus type 1 J Infect Dis 2002 185 456 462 10.1086/338572 11865397

[B40] Tsubakimoto A Saito I Mannami T Naito Y Nakamura S Dohi Y Yonemasu K Impact of metabolic syndrome on brachial-ankle pulse wave velocity in Japanese Hypertens Res 2006 29 29 37 10.1291/hypres.29.29 16715651

[B41] Tomiyama H Koji Y Yambe M Motobe K Shiina K Gulnisa Z Yamamoto Y Yamashina A Elevated C-reactive protein augments increased arterial stiffness in subjects with the metabolic syndrome Hypertension 2005 45 997 1003 10.1161/01.HYP.0000165018.63523.8a 15837828

[B42] Reilly MP Rader DJ The metabolic syndrome: more than the sum of its parts? Circulation 2003 108 1546 1551 10.1161/01.CIR.0000088846.10655.E0 14517150

[B43] Torriani FJ Komarow L Parker RA Cotter BR Currier JS Dube MP Fichtenbaum CJ Gerschenson M Mitchell CK Murphy RL Squires K Stein JH Endothelial function in human immunodeficiency virus-infected antiretroviral-naive subjects before and after starting potent antiretroviral therapy: The ACTG (AIDS Clinical Trials Group) Study 5152s J Am Coll Cardiol 2008 52 569 576 10.1016/j.jacc.2008.04.049 18687253 PMC2603599

